# Supplementary material for: An open-source framework for large-scale, flexible evaluation of biomedical text mining systems
Source: J Biomed Discov Collab. 2008 Jan 29;3:1. doi: 10.1186/1747-5333-3-1 (PMC2276192; doi:10.1186/1747-5333-3-1)
Supplement: Additional file 1 — Evaluation platform source code. The additional file contains the software evaluation framework discussed in this paper. The most current release can be downloaded from SourceForge.net [1]. See the accompanying README file for instructions on installation and use. Also included in the distribution is a collection of UIMA wrappers for some commonly used BioNLP tools and annotated corpora. [file 1747-5333-3-1-S1.zip › bionlp_uima_repository-1.0.1/docs/api/src-html/edu/uchsc/ccp/uima/util/opendmap/TestSummarizer.html]

```
001    /**
002     * The OpenDMAP for Protege Project
003     * February 2006
004     */
005    package edu.uchsc.ccp.uima.util.opendmap;
006    
007    import java.util.ArrayList;
008    import java.util.HashMap;
009    import java.util.Iterator;
010    
011    import org.apache.uima.jcas.JCas;
012    
013    import edu.uchsc.ccp.uima.annotation.CCPTextAnnotation;
014    import edu.uchsc.ccp.uima.mention.CCPClassMention;
015    
016    /**
017     * This class prints out selected contents of the CAS in the standard
018     * annotation summery format.
019     * 
020     * @author R. James Firby
021     */
022    public class TestSummarizer {
023            
024            private static final String MENTION_SEPARATOR = ";";
025            private static final String SLOT_SEPARATOR = ",";
026            
027            /* The mentions in the CAS to print */
028            private HashMap<String, String[]> mentionsOfInterest = null;
029            
030            /**
031             * Create a new TestSummarizer that will generate summary strings for the mentions and slots
032             * specified in the pattern.
033             * <p>
034             * A mention pattern has the form:
035             * <pre>
036             *   type,slot,slot;type,slot,slot;...
037             * </pre>
038             * Where 'type' is the name of a mention type to print, and the 'slot's
039             * following the 'type' are thje slots for that type to include in the
040             * output.
041             * 
042             * @param mentionPattern The pattern defining the mention types and slots to print.
043             */
044            public TestSummarizer(String mentionPattern) {
045                    if (mentionPattern != null) {
046            String mentionTypes[] = mentionPattern.split(MENTION_SEPARATOR);
047            if ((mentionTypes != null) && (mentionTypes.length > 0)) {
048                    mentionsOfInterest = new HashMap<String, String[]>();
049                    for (int i=0; i<mentionTypes.length; i++) {
050                            String typeParts[] = mentionTypes[i].split(SLOT_SEPARATOR);
051                            if ((typeParts != null) && (typeParts.length > 0)) {
052                                    String typeName = typeParts[0].trim().toLowerCase();
053                                    if (typeParts.length > 1) {
054                                            String slotNames[] = new String[typeParts.length-1];
055                                            for (int j=1; j<typeParts.length; j++)
056                                                    slotNames[j-1] = typeParts[j].trim();
057                                            mentionsOfInterest.put(typeName, slotNames);
058                                    } else {
059                                            mentionsOfInterest.put(typeName, null);
060                                    }
061                            }
062                    }
063            }
064        }
065            }
066            
067            /**
068             * Generate summary strings for all annotations in the JCAS that are described in the
069             * pattern string for this summarizer.
070             * 
071             * @param docId The document ID that has been analyzed to fill the JCas
072             * @param jcas The JCas holding the annotations to be summarized
073             * @return A set of summary strings, one for each annotation of interest in the JCas
074             */
075            public ArrayList<String> summarizeJCas(String docId, JCas jcas) {
076                    ArrayList<String> summary = new ArrayList<String>();
077                    // Get the GeneRIF text
078                    String docText = jcas.getDocumentText();
079              // Print out all output annotations for this generif
080              Iterator annotationIter = jcas.getJFSIndexRepository().getAnnotationIndex(CCPTextAnnotation.type).iterator();
081              while ((annotationIter != null) && annotationIter.hasNext()) {
082                    Object thing = annotationIter.next();
083                    if (thing instanceof CCPTextAnnotation) {
084                            CCPTextAnnotation annotation = (CCPTextAnnotation) thing;
085                            if (isAnnotationOfInterest(annotation)) {
086                                    // Get the annotation string and print it
087                                    AnnotationString annotationString = new AnnotationString(annotation, slotsOfInterest(annotation), docId, docText);
088                                    summary.add(annotationString.toString());
089                            }
090                    }
091              }
092              // Done
093              return summary;
094            }
095            
096            /**
097             * Checks whether and annotation is specified in the pattern string for
098             * this summarizer.
099             * 
100             * @param annotation The annotation to check.
101             * @return 'True' if this annotation should be summarized in the output.
102             */
103      private boolean isAnnotationOfInterest(CCPTextAnnotation annotation) {
104            // If nothing was specified, default to everything
105            if (mentionsOfInterest == null) return true;
106            // See if this annotations mention is of interest
107            CCPClassMention mention = annotation.getClassMention();
108                    String className = mention.getMentionName();
109                    return ((className != null) && mentionsOfInterest.containsKey(className.trim().toLowerCase()));
110      }
111      
112      /**
113       * Get all the slots that should be included in the summary for this annotation.
114       * 
115       * @param annotation The annotation to summarize.
116       * @return The set of slots to include in the summary.
117       */
118      private String[] slotsOfInterest(CCPTextAnnotation annotation) {
119            // If nothing was specified, default to everything
120            if (mentionsOfInterest == null) return null;
121            // See if this annotations mention is of interest
122            CCPClassMention mention = annotation.getClassMention();
123                    String className = mention.getMentionName();
124                    if (className != null) {
125                            return mentionsOfInterest.get(className.trim().toLowerCase());
126                    } else {
127                            return null;
128                    }
129            }
130    
131    }
```
